# Serologic Status of *Borrelia burgdorferi* sensu lato in Patients with Cardiovascular Changes

**DOI:** 10.3390/ijerph20032239

**Published:** 2023-01-27

**Authors:** Katarzyna Pietruszka, Farbod Reagan, Janusz Stążka, Małgorzata M. Kozioł

**Affiliations:** 1 Students Scientific Association at the Chair and Department of Medical Microbiology, Medical University of Lublin, 20-093 Lublin, Poland; 2 Chair and Department of Medical Microbiology, Medical University of Lublin, 20-093 Lublin, Poland; 3 Department of Cardiac Surgery, Medical University of Lublin, 20-093 Lublin, Poland

**Keywords:** anti-*Borrelia*, seroprevalence, cardiovascular disease, CABG, Lyme carditis

## Abstract

Cardiovascular diseases, particularly coronary heart disease (CHD) caused by atherosclerosis, have the highest worldwide incidence and mortality rate of any type of disease. Aside from risk factors associated with lifestyle and comorbidities, infectious agents such as *Borrelia burgdorferi* sensu lato spirochetes, which cause Lyme disease, may also play a role in the development of cardiovascular disease. A growing number of scientific papers have mentioned Lyme carditis. The aim of this study was to find the level of anti-*Borrelia* IgG antibodies in the blood serum of patients with advanced coronary heart disease. Materials and methods: The study group included 70 patients undergoing coronary artery bypass grafting (CABG) and off-pump coronary artery bypass (OPCAB) surgery aged 50 to 82 (average 68.26). The ELISA test was used to detect anti-*Borrelia*/IgG antibodies in the blood serum. Serological testing revealed seropositivity in 34.29% of patients and ‘borderline results’ in 17.14% of patients. We found a link between antibody levels and tick bites but not with other risk factors for the development of CHD. Conclusions: These findings support the idea that, as one of many factors, the contact with spirochetal antigens may indicate a potential positive correlation with the formation of cardiovascular changes. More research, not only at the diagnostic level but also at the advanced research level, is needed.

## 1. Introduction

Cardiovascular diseases (CVD) are a group of chronic diseases of a heterogeneous nature with the highest worldwide incidence and mortality rate of any type of disease. The most common cardiovascular disorders include coronary heart disease (CHD) caused by atherosclerosis and cerebrovascular disease. Currently, it is estimated that approximately 30% of deaths worldwide are caused by cardiovascular diseases [1,2,3]. In Europe, the incidence is high, with about 4 million deaths per year. The elderly over 65 are particularly exposed to the development of cardiological changes. It has been observed that the incidence and mortality rate from cardiovascular diseases are higher in women than in men [2,3,4]. Cardiovascular diseases progress gradually, and their course may be asymptomatic. The appearance of symptoms in CHD adversely affects the body and relatively reduces quality of life [1,2,3,4,5]. The results demonstrate a relationship between early-stage atherosclerosis and a higher prevalence of ischemic heart disease and, consequently, the occurrence of complications including symptomatic angina, myocardial infarction, and death from coronary lesions [1,5]. Due to the high risk of cardiovascular complications and the inability to completely cure certain disease entities, surgical intervention is often required.

The occurrence of the disease is influenced by many factors, including so-called modifiable and non-modifiable factors (e.g., age, gender, and positive family history of CVD). It is believed that in most cases more than one factor contributes to the development of the disease. The human-dependent factors include overweight, eating habits, elevated blood cholesterol, triglycerides, smoking, alcohol consumption, and low physical activity [1,6]. In addition to so many variables, it is worth paying attention to whether the changes in the coronary vessels are also related to previous infections. It is speculated that various infectious agents, both bacterial and viral, are also responsible for cardiological changes in the coronary arteries. The development of infection in the vessel walls may accelerate the process of atherosclerotic plaque formation. It is concluded that viral agents such as cytomegalovirus (CMV) and hepatitis C virus (HCV) may also be involved in the changes. Infections with bacterial pathogens, such as *Mycoplasma pneumoniae* and *Chlamydia pneumoniae*, have a potentially positive correlation with the formation of cardiological changes [7,8]. Spirochetes *Borrelia burgdorferi* sensu lato (Bbsl), which are etiological agents of Lyme disease (LD), are also suspected of causing some cardiological changes [7,8,9]. LD is a vector disease in which bacteria are transmitted to the human body by an infected tick bite. It is a multi-organ disease that, due to the various locations of spirochetes in the human body, can mimic many diseases. Pathogens can cause mild to severe skin lesions, and they frequently attack the osteoarticular system or cause disorders of the nervous system, both peripheral and central. In addition, other organs may be damaged, and infection may cause subtle cardiac changes [10]. In the disseminated phase, which develops over weeks or months after infection, the bacteria migrate from the infected skin to the blood and lymph vessels, through which they spread to various organs. The infection can cause a variety of symptoms depending on the final bacterial location. As a result of spirochete dissemination, the initial symptoms of Lyme disease may appear resulting from joint, neurological, and cardiac disorders [10,11]. The aim of this study, in terms of potential cardiac changes, was to assess the levels of anti-*Borrelia* IgG antibodies in the blood of patients with advanced coronary artery disease.

## 2. Materials and Methods

### 2.1. Study Group

The study group consisted of 70 patients: 22 women (31.43%) and 48 men (68.57%), with a mean age of 68.26 years (50–82), who required surgical treatment for coronary artery disease. The patients underwent a cardiac surgical procedure at the Department of Cardiac Surgery at the Medical University in Lublin, Poland: coronary artery bypass grafting—CABG (63/90%), and off-pump coronary artery bypass—OPCAB (7/10%) procedure. The operations were elective (43/61.43%) and urgent (27/38.57%), mostly due to unstable coronary artery disease. The EuroSCORE scale (European System for Cardiac Operative Risk Evaluation) was used to assess the group, and risk factors such as BMI, diabetes, arterial hypertension, arrhythmias, hypercholesterolemia, smoking, chronic lung diseases, and a history of pneumonia were collected. The patients were interviewed about Lyme disease (a potential tick bite risk), the occurrence of a noticeable tick bite during their lifetime, and the occurrence of typical Lyme disease symptoms.

### 2.2. Laboratory Testing

Whole blood samples were collected and centrifuged. The serum was separated and stored at −80 °C until it was assayed. The anti-*Borrelia* IgG ELISA test was used to determine the IgG concentration (Euroimmun, Lübeck, Germany). The product was used according to the manufacturer’s instructions. Anti-*Borrelia* interpretation ranges: positive ≥ 22 RU/mL; borderline ≥16 to <22 RU/mL; negative < 16 RU/mL.

### 2.3. Statistical Analysis

The collected data were statistically analyzed using Statistica v.12.5 (StatSoft).

## 3. Results

The analysis included 70 people who had undergone an invasive procedure to treat coronary artery disease. According to the patient’s declaration, the first symptoms of the disease appeared most often in the previous 1–5 years—35.71% (the remaining ones: within a year—24.29%; in the previous 5–10 years—15.71%; over 10 years—24.29%). The mean EuroSCORE scale (European System for Cardiac Operative Risk Evaluation) was 1.83 (estimated low risk of cardiac surgery treatment), and the mean BMI was 29.27 (overweight) ± 4.19. Men (68.57%) predominated in the study group, as did hypertension (80%) and nonsmokers (70%). Diabetes was found in 41.43% of the patients who underwent an operation. Detailed characteristics of the group are presented in Table 1.

The patients were interviewed about Lyme disease. An unfortunate limitation of our study was that we collected data from only some of the patients (20% of the study group). The obtained data, in correlation with the results of laboratory tests, showed significance in terms of a noticed tick bite (*p* = 0.05898 *).

Seropositivity against *Borrelia* spirochetes was detected in 34.29% of the patients. Detailed results of the concentration of anti-*Borrelia* (IgG) antibodies in the tested samples are presented in Table 2.

## 4. Discussion

Coronary heart disease is now one of the leading causes of morbidity and mortality worldwide. In this case, vascular changes include atherosclerosis of blood vessels made of lipids and connective tissue. Atherosclerosis is marked by focal, asymmetrical occurrence and as a result, an inflammatory process begins in the pathologically changed vessels, leading to endothelial dysfunction. Atherosclerotic plaque rupture and acute coronary syndromes (ACS) can occur in the advanced stages. ACS complications can even lead to heart failure [5,12]. Due to the complexity of the pathogenic process, patients are admitted to the cardiosurgical units and may require invasive procedures. The condition of the patients is integrally associated with multiple concomitant diseases and with the presence of risk factors leading to vascular changes, and consequently with ischemic heart disease. Many of the risk factors have been identified so far. According to our study, a significant portion of our patients had diabetes (41.43%), were overweight or obese (average BMI = 29.27), and had hypercholesterolemia (35.71%). Additionally, the patients acknowledged having low levels of physical activity, which may have been a result of their advanced age (the average age of the patients was 68.26 years) and the disease progression itself. According to their findings, Ostovan et al. [13], in agreement with other researchers, stated that patients undergoing CABG are overweight, have high cholesterol levels, and are treated for hypertension and diabetes. Moreover, the patients frequently smoked actively (33.7%) [13]. Smoking is a substantial risk factor; however, in our study group, it was not statistically significant (patients who did not smoke accounted for 70% of the group). A study by McAlister et al. [14] showed that one of the main diseases accompanying patients undergoing CABG is type II diabetes, which affected 95% of the patients. In our research, only 42% of the patients had diabetes.

In addition to looking for the underlying causes of coronary artery disease (CAD), other variables, such as infectious ones, are being considered. Because the atherosclerotic process is so complex, some experts believe that microbial elements may be involved in accelerating the formation of plaques in the arteries. *Mycoplasma pneumoniae* and *Chlamydia pneumoniae*, two bacterial infections linked to atypical pneumonia, are among the probable CAD pathogens [7]. The collected data showed that 62.82% of the patients recalled having lower respiratory tract infections in the past, sometimes more than once and with varying severity. The majority of the participants also admitted to using antibiotics for this reason. It should be noted that few of the participants recalled having an infection because the microorganisms did not cause typical signs of infection [15]. An examination of the immune response to these etiological factors would give a complete picture of the past infection.

*B. burgdorferi* s.l. is also considered to be a potential risk factor when it comes to changes in the cardiovascular system. It is said to be so-called Lyme carditis (LC), a disease primarily associated with cardiac conduction [16]. Changes occurring within the heart are often defined as disturbances in the conduction of electrical impulses. It is estimated that this abnormality occurs in approximately 4 to 8% of patients with untreated Lyme disease and is one of the causes of cardiac death. These disorders have been found to be three times more common in men than in women. An inflammatory reaction develops as a result of bacterial spread in the body via the blood, which can involve all the layers of the heart muscle [16,17,18]. Heart problems usually appear a few weeks or a few months after infection. Symptoms include loss of consciousness, dizziness, palpitations, chest pain, and shortness of breath [11]. The most common symptoms are conduction disorders (which manifest as various degrees of heart block), atrial fibrillation (AFib), and tachycardia. Less common are myocarditis, pericarditis, endocarditis, and valvular insufficiency [19]. Atrioventricular block (AV) is a typical symptom of Lyme carditis caused by a temporary inhibition or slowdown in the transmission of impulses from the atria to the ventricles. It is estimated that cardiac changes in about 90% of patients are due to a high degree of AV block. If LD is not properly treated or undiagnosed, the third degree of AV can lead to sudden cardiac death due to ventricular fibrillation (VF) or ventricular tachycardia (VT) [20,21]. Myocarditis is another disease caused by spirochete infection. It is characterized by a mild and short-term course, but it can progress to hypertrophic cardiomyopathy (HCM), congestive heart failure (CHF), or left ventricular (LV) dysfunction. This abnormality is not very common; it is estimated to occur in 0.4% to 4% of patients infected with *Borrelia burgdorferi* s.l. in Europe [20].

Moreover, *B. burgdorferi* s.l. may lead to changes in arteries, and CAD as a result. An inflammatory condition known as atherosclerosis is associated with artery plaque formation. Plaques have been found to include a variety of bacteria [22]. Völzke et al. [23] concluded that exposure to infectious pathogens such as spirochetes increases the risk of atherosclerosis in tick-endemic areas.

Lyme disease is a multi-system disease that affects approximately 20,000 people each year in Poland and 476,000 in the United States. LD is often called “the great imitator” because its clinical symptoms are nonspecific [24,25]. Joint pains or flu-like symptoms, particularly in the elderly, are frequently ignored, which postpones diagnosis of the illness. Increased morbidity of LD is observed in tick-endemic regions, which include the patients in our study, where there is a significant percentage of people with coronary artery disease. Only a few patients undergoing CABG were interviewed about LD in our study. A tick-biting event had previously occurred in 57.14 percent of the patients, according to the data gathered, but they had not been diagnosed or treated accordingly. Most of the patients revealed no erythema-like changes during their lifetime (85.72% vs. 14.28%). Research carried out in Sweden confirmed that over 50% of the study group usually do not remember symptoms indicating Lyme disease or episodes of tick bite [26].

Diagnosis of *B. burgdorferi* s.l. infection is determined depending on the onset of clinical symptoms. Erythema migrans, often known as EM, is a characteristic lesion that at first might be mistaken for a reaction to being bitten by an insect, for example. In the absence of visible clinical manifestation, diagnosis is difficult due to nonspecific symptoms and the patient’s lack of awareness of a potential tick bite. In the event that the infection is not treated, bacteria can spread to other parts of the body [27]. There are numerous methods for identifying spirochetes; however, not all of them are recommended [28]. Molecular techniques, which use PCR (polymerase chain reaction), have some limitations and should be considered as a supplementary method. One of the issues is the possibility of contamination during diagnostics; the genetic material found may result from the spirochete’s bacterial cells rupturing, or it may be the consequence of an infection that is dormant [20]. The advised method is assessing the immune response to the infection, although there are time limitations in identifying early infection (seroconversion). Certain antibodies, such as immunoglobulins class M (IgM) and class G (IgG), can be detected using commercially available tests. These tests look for antibodies in the patient’s blood serum as well as, depending on the clinical manifestation, in the cerebrospinal fluid. The serologic diagnosis uses a two-step approach. It is based on a screening test (Enzyme-Linked Immunosorbent Assay (ELISA)) which is highly sensitive and provides estimated information about the quantification of anti-*Borrelia* antibodies. Positive or equivocal results must be confirmed by the immunoblot technique. In order to start pharmacological treatment of patients with elevated immunoglobulin, the results of the serological tests should be correlated with the medical history and clinical condition of the patient due to the high genetic variability and differences between individual *Borrelia* genospecies [20,27,28,29]. When attempting to diagnose Lyme carditis, it is essential to identify any changes that have taken place in the heart and to confirm the infection with serological testing [29].

Screening for anti-*Borrelia* IgG seropositivity performed in cardiac surgery patients showed that elevated antibody level results, interpreted as positive, were obtained in 34.29%. It is likely that the patients had contact with ticks or spirochete antigens. Moreover, the applied antibiotic therapy for pneumonia could possibly affect the elimination of the potential infection and lower the concentration of antibodies. Extensive research by Völzke et al. [23] on a randomized group of patients (2483 people) showed that seropositivity in the ELISA test was observed in 108 patients (4.3%). The results appear to indicate that spirochete infection was an independent factor in the development of carotid atherosclerosis. However, an analysis of the data in connection with other parameters (age, sex, atherosclerosis risk factors, and others) in multivariate statistical models indicated that the presence of antibodies was significant in the progression of the disease [23]. It is challenging to locate comparable studies in the literature. Although more and more studies emphasize the role of spirochetes in the development of typical LC, the interest in the participation of spirochetes in the aspect of cardiac changes in cardiac surgery patients has been inappreciable so far. The obtained results encourage extending the research to a larger group of patients.

## 5. Conclusions

*Borrelia burgdorferi* s.l. infections are widespread in the population, and diagnosis of the disease is difficult due to the lack of specific symptoms in many cases. Infected people do not remember the bite episodes, so they are often unaware of a potential infection. According to the findings of our study, more than a third of the patients had elevated IgG levels against *Borrelia* as detected by a screening test, indicating previous contact with spirochaetes. We are aware of the limitations of our results, but these findings can indicate a new direction in the study of the development of cardiological alterations and the onset of coronary artery disease. Furthermore, extensive research is necessary, both at the diagnostic level and with advanced research techniques.

## Figures and Tables

**Table 1:** General data/risk factors of the study group and assessment of its significance in correlation with the performed laboratory tests.

Study Group Characteristics			n	%	Test *p*
cigarette smoker		current	3	4.29	0.88669
		in the past	18	25.71	
		never	49	70	
diabetes			29	41.43	0.00664
hypertension			56	80	0.15460
COPD (Chronic obstructive pulmonary disease)			2	2.85	0.11105
Asthma			1	1.43	0.33811
hyperlipidemia			25	35.71	0.64101
recently had a heart attack			12	17.14	0.83185
procedure	elective		43	61.43	0.19644
	Semi-elective		27	38.57	
History of pneumonia/bronchitis			44	62.86	0.38125

**Table 2:** Laboratory findings of Anti-*Borrelia* IgG levels with interpretation provided in the kit (Euroimmun, Germany).

Anti-*Borrelia* ELISA (IgG)			
N = 70	positive (≥22 RU/mL)	borderline (≥16 to <22 RU/mL)	negative (<16 RU/mL)
	24 (34.29%)	12 (17.14%)	34 (48.57%)
Anti-*Borrelia* RU/mL	av. 103.615 (23.3–293.86) (SD 77.9)	av. 19.5078 (16.25–21.38) (SD 1.52)	av. 9.61044 (2.181–15.77) (SD 3.83)

SD—standard deviation.

## Data Availability

The datasets used and/or analyzed in the current study are available from the corresponding author on reasonable request.
